# Deep Learning-Based Acute Ischemic Stroke Lesion Segmentation Method on Multimodal MR Images Using a Few Fully Labeled Subjects

**DOI:** 10.1155/2021/3628179

**Published:** 2021-01-29

**Authors:** Bin Zhao, Zhiyang Liu, Guohua Liu, Chen Cao, Song Jin, Hong Wu, Shuxue Ding

**Affiliations:** ^1^Tianjin Key Laboratory of Optoelectronic Sensor and Sensing Network Technology, College of Electronic Information and Optical Engineering, Nankai University, Tianjin 300350, China; ^2^Key Laboratory for Cerebral Artery and Neural Degeneration of Tianjin, Department of Medical Imaging, Tianjin Huanhu Hospital, Tianjin 300350, China; ^3^School of Artificial Intelligence, Guilin University of Electronic Technology, Guilin Guangxi 541004, China

## Abstract

Acute ischemic stroke (AIS) has been a common threat to human health and may lead to severe outcomes without proper and prompt treatment. To precisely diagnose AIS, it is of paramount importance to quantitatively evaluate the AIS lesions. By adopting a convolutional neural network (CNN), many automatic methods for ischemic stroke lesion segmentation on magnetic resonance imaging (MRI) have been proposed. However, most CNN-based methods should be trained on a large amount of fully labeled subjects, and the label annotation is a labor-intensive and time-consuming task. Therefore, in this paper, we propose to use a mixture of many weakly labeled and a few fully labeled subjects to relieve the thirst of fully labeled subjects. In particular, a multifeature map fusion network (MFMF-Network) with two branches is proposed, where hundreds of weakly labeled subjects are used to train the classification branch, and several fully labeled subjects are adopted to tune the segmentation branch. By training on 398 weakly labeled and 5 fully labeled subjects, the proposed method is able to achieve a mean dice coefficient of 0.699 ± 0.128 on a test set with 179 subjects. The lesion-wise and subject-wise metrics are also evaluated, where a lesion-wise F1 score of 0.886 and a subject-wise detection rate of 1 are achieved.

## 1. Introduction

Stroke has been one of the most serious threats to human health, which can lead to long-term disability or even death [1]. In general, stroke can be divided into ischemia and hemorrhage based on the types of cerebrovascular accidents, where ischemic stroke accounts for 87% [2]. In clinical practice, multimodal magnetic resonance images (MRIs), including the diffusion-weighted imaging (DWI) and the apparent diffusion coefficient (ADC) maps derived from multiple DWI images with different *b* values, have been used in diagnosing acute ischemic stroke (AIS), thanks to the short acquisition time and high sensitivity [3]. As AIS progresses rapidly and may lead to severe outcomes, it is of paramount importance to quickly diagnose and quantitatively evaluate the AIS lesions from the multimodal MRIs, which is, however, time-consuming and requires experienced medical imaging clinicians. Therefore, it is quite necessary to develop automatic methods in analyzing the images.

Many automatic stroke lesion segmentation methods have been developed in the literature. For instance, Nabizadeh et al. [4] proposed a gravitational histogram optimization by identifying the abnormal intensity. To reduce the false positive rate, Mitra et al. [5] used the random forest to extract features and identify the lesions based on multimodal MRIs. Maier et al. [6] adopted the support vector machine based on the local features extracted from multimodal MRIs. Although such methods achieved high performance on ischemic stroke lesion segmentation, their modeling capabilities were significantly limited due to their heavy dependence on handcrafted features.

**Figure 1:**
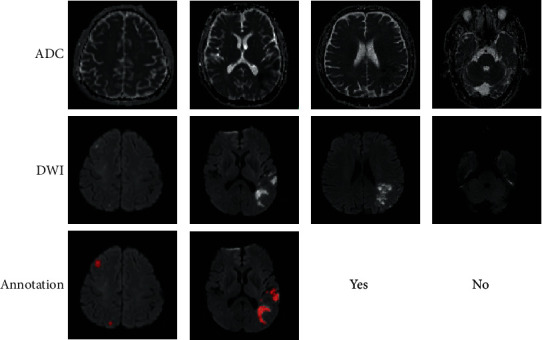
Examples of fully labeled and weakly labeled subjects. The first two columns show fully labeled examples, and the last two are weakly labeled ones, where the label “yes” indicates that the slice has a lesion and “no” indicates the opposite. Best viewed in color.

A convolutional neural network (CNN) has recently presented an exceptional performance in computer vision. By training on a large number of fully labeled subjects where the stroke lesions were annotated in a pixel-by-pixel manner, the CNN-based methods have shown their great potentials in segmenting ischemic stroke lesions on the MRIs [7–11]. As a CNN typically has millions of parameters, such methods require hundreds of fully labeled subjects to train the CNN.  Figure 1 presents some examples of fully labeled subjects. It is obvious that annotating pixel-by-pixel labels is a tedious task and would take a significant amount of time to establish a large dataset with fully labeled subjects, which makes it impossible to establish a medical imaging dataset with a comparable size to the commonly used datasets in computer vision. This motivates us to develop segmentation methods while reducing the annotation burden for medical imaging clinicians.

Few-shot learning has recently been adopted in image semantic segmentation [12–15]. By fine-tuning the network parameters with a few samples, the CNN can achieve high segmentation accuracy in many tasks. Typically, the few-shot learning methods require ImageNet [16] pretrained parameters to help extract features. In the medical image segmentation task, however, it is not possible to find a dataset as large as ImageNet to obtain pretrained parameters. Therefore, it is necessary to design an auxiliary task with easily obtained labels to pretrain the network.

In particular, we make use of many weakly labeled subjects and propose to use weakly supervised learning method to facilitate the AIS lesion segmentation. Different from the other AIS lesion segmentation methods [17–21], the weakly labeled subjects are annotated as whether each slice of a subject incorporates lesion or not, as shown in Figure 1, which significantly reduces the cost on annotation.

Our proposed method consists of three processes: classification, segmentation, and inference. In the classification process, the network is trained on the weakly labeled subjects as a classifier to obtain a set of pretrained parameters. In the segmentation process, the network freezes the pretrained parameter and is further trained on the fully labeled subjects. In the inference process, the classification branch generates class activation mapping (CAM) [22] and the segmentation branch predicts the segmentation result. A postprocessing algorithm is adopted to combine the CAM with the segmentation result to generate a final prediction. By using 398 weakly labeled subjects and 5 fully labeled ones, the proposed method is able to achieve a dice coefficient of 0.699 ± 0.128. The lesion-wise and subject-wise performances are also evaluated, where a lesion-wise F1 score of 0.886 and a subject-wise detection rate of 1 are achieved.

## 2. Materials and Methods

**Figure 2:**
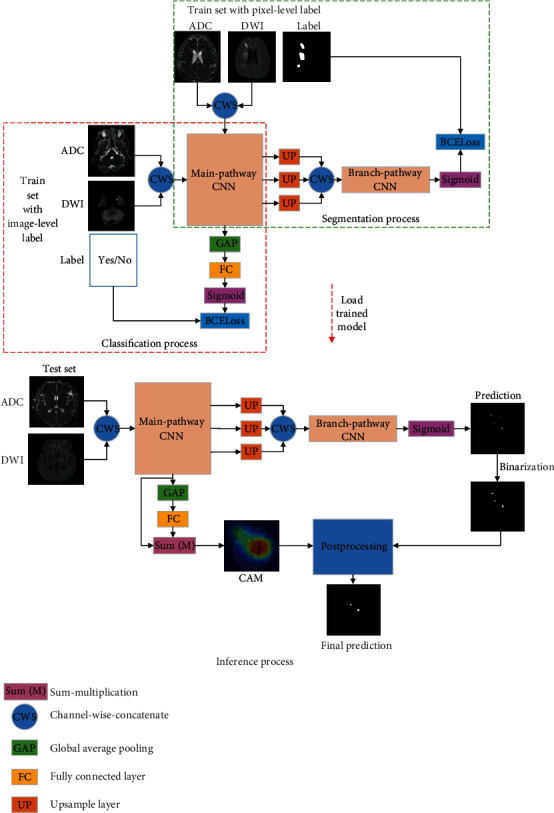
Whole pipeline of the proposed method. Best viewed in color.

In this section, we propose a deep learning-based method using a few fully labeled subjects for AIS segmentation on two-modal MR images, and the pipeline is presented in Figure 2. In particular, our proposed method consists of three processes: classification, segmentation, and inference. In the classification process, the network is trained on the weakly labeled subjects as a classifier. This process obtains a set of pretrained parameters. In the segmentation process, the network is trained end-to-end on the fully labeled subjects by freezing the pretrained parameters. That is to say, in order to avoid overfitting, only the decoder is trained using a few fully labeled subjects. In the inference process, the classification branch generates class activation mapping (CAM) [22] and the segmentation branch predicts the segmentation result. Then, a postprocessing method is adopted to combine the CAM with the segmentation result to generate a final prediction. As we will show in this paper, only 5 fully labeled subjects are adequate to achieve accurate segmentation.

### 2.1. Multifeature Map Fusion Network

**Figure 3:**
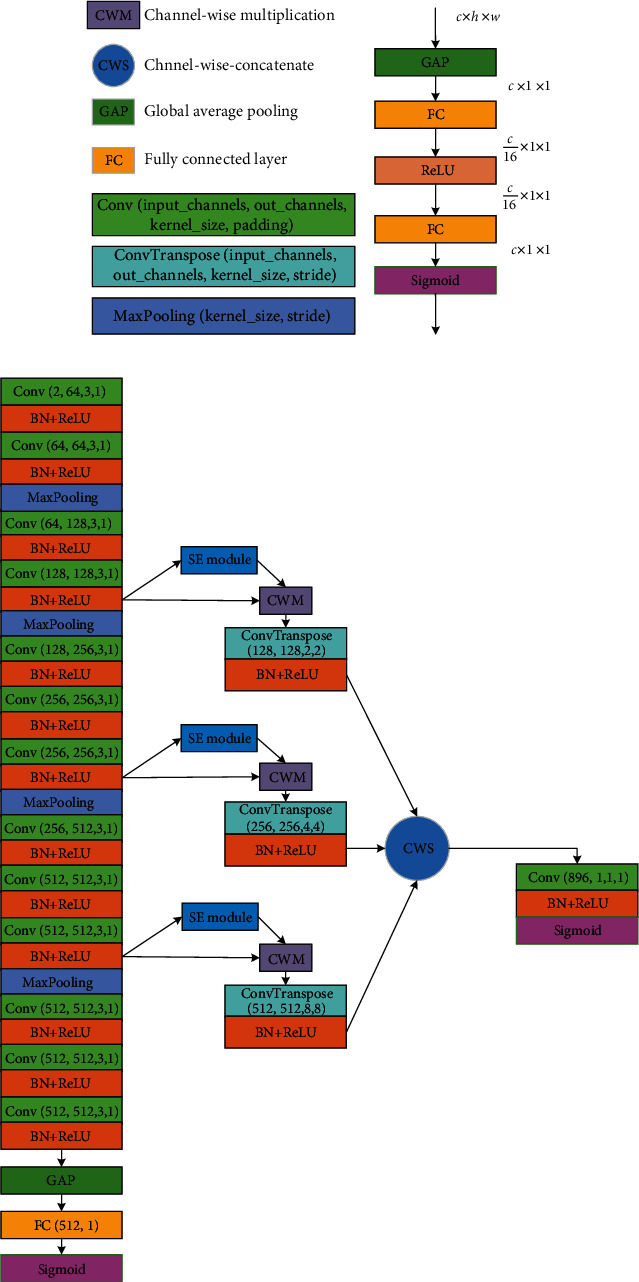
Our proposed network architecture. (a) Unit parameter description. (b) SE module. (c) Multifeature map fusion network (MFMF-Network). Best viewed in color.

Different from the few-shot semantic segmentation on natural images where the ImageNet pretrained parameters were easily obtained, there is no available large dataset for brain MRIs. A multifeature map fusion network (MFMF-Network) is proposed and trained on the weakly labeled subjects to extract features whose architecture is presented in Figure 3. The proposed MFMF-Network is a two-branch CNN, where the backbone CNN is a VGG16 [23] truncated before the 5th MaxPooling layer.

As Figure 2 shows, we add a global average pooling (GAP) followed by a fully connected (FC) layer at the top of the main-pathway CNN as the classification branch, which is trained by the weakly labeled subjects at the classification process. On the other hand, the segmentation branch fuses the upsampled feature maps from convolutional blocks 4, 7, and 10, which is used to generate a pixel-wise segmentation map.

Intuitively, the feature maps of the deeper convolutional block have much lower spatial resolution than the original input images but with better semantic information. We further incorporate the squeeze-and-excitation (SE) module [24] into the upsample layer as depicted in Figure 3(b), such that the network can focus on the feature maps that contribute most to AIS segmentation.

The training of the MFMF-Network takes two steps. In the classification process, the backbone CNN, together with the classification branch, is trained on the weakly labeled subjects as a classifier. In the segmentation process, the segmentation branch is trained on a few fully labeled subjects, while the parameters of the backbone CNN are frozen.

### 2.2. Postprocessing

In the inference process, as Figure 2 shows, the classification branch generates CAM [22] as

(1) $$\begin{array}{c} M_{c}\left(x,y\right)=\underset{k}{\sum }w_{k}^{c}∙f_{k}\left(x,y\right), \end{array}$$

where *f*_*k*_(*x*, *y*) represents the activation of unit *k* in the last convolutional layer of main-pathway CNN at the spatial location (*x*, *y*) and *w*_*k*_ is the weight corresponding to the class *c* for unit *k*. Note that as the AIS lesion segmentation is a binary segmentation task, that is, *c* = 2, therefore, we only consider the CAM of the lesion class. The CAM is normalized to generate a segmentation probability map, and a binary segmentation result *M*_*c*_(*x*, *y*; *δ*) is further obtained by using a threshold of *δ* = 0.5. Simultaneously, the segmentation branch predicts the segmentation probability map. The binary segmentation result *S*_*c*_(*x*, *y*; *δ*) at the spatial location (*x*, *y*) is also obtained by using the same threshold *δ*.

Nevertheless, since few fully labeled subjects are used to train the segmentation branch, it is inevitable to generate some false positives. To fully utilize the rich semantic information from the weakly labeled data, we further fuse the CAM generated from the classification branch with the segmentation branch output to reduce the FPs, which is computed as

(2) $$\begin{array}{c} P_{c}\left(x,y\right)=M_{c}\left(x,y;\delta \right)∙S_{c}\left(x,y;\delta \right). \end{array}$$

### 2.3. Evaluation Metrics

In this subsection, we introduce a number of metrics to evaluate our proposed method. First, the dice coefficient (DC) is used to evaluate the pixel-level segmentation performance. It measures the overlap between the predicted segmentation *P* and the ground truth *G* and is formulated as

(3) $$\begin{array}{c} \text{DC}=\frac{2\left|G\cap P\right|}{\left|G\right|+\left|P\right|}, \end{array}$$

where ∣∙∣ denotes the number of pixels in the set.

In addition, we further propose the lesion-wise precision rate *P*_L_, the lesion-wise recall rate *R*_L_, and the lesion-wise F1 score as metrics, which are defined as

(4) $$\begin{array}{c} P_{\text{L}}=\frac{\text{m}\#\text{TP}}{\text{m}\#\text{TP}+\text{m}\#\text{FP}}, \end{array}$$

(5) $$\begin{array}{c} R_{\text{L}}=\frac{\text{m}\#\text{TP}}{\text{m}\#\text{TP}+\text{m}\#\text{FN}}, \end{array}$$

(6) $$\begin{array}{c} \text{F}1=\frac{2P_{\text{L}}∙R_{\text{L}}}{P_{\text{L}}+R_{\text{L}}}, \end{array}$$

where m#TP, m#FP, and m#FN are the mean number of true positives (TPs), false positives (FPs), and false negatives (FNs), respectively, which are calculated in a lesion-wise manner. In this paper, a 3D connected component is performed on both the ground truth and the predicted segmentation map. A TP is defined as a connected region on the predicted segmentation map that overlaps with that on the ground truth. The number of TPs is counted on each subject, and the mean number of TPs (m#TP) is then obtained by averaging the number of TPs over all subjects. A FP is counted if a region on the predicted segmentation has no overlap with any region on the ground truth. While a FN is counted if a region on the ground truth has no overlap with any region on the predicted segmentation.

We further use the detection rate (DR) to measure missed subjects as a subject-wise metric, which is defined as

(7) $$\begin{array}{c} \text{DR}=\frac{N_{\text{TP}}}{N}, \end{array}$$

where *N* denotes the number of all subjects and *N*_TP_ denotes the number of subjects with any TP lesion detection.

## 3. Experiments

In this section, we will introduce the experimental data, the implementation details, and the results.

### 3.1. Data and Preprocessing

The experimental data includes 582 subjects with AIS lesions, which were collected from a retrospective database of Tianjin Huanhu Hospital (Tianjin, China) and anonymized prior to the use of researchers. Ethical approval was granted by the Tianjin Huanhu Hospital Medical Ethics Committee. MR images were acquired from three MR scanners, with two 3T MR scanners (Skyra, Siemens, and Trio, Siemens) and one 1.5T MR scanner (Avanto, Siemens). DWIs were acquired using a spin echo-type echo planner imaging (SE-EPI) sequence with *b* values of 0 and 1000 s/mm^2^. The parameters used in DWI acquisition are shown in Table 1. ADC maps were calculated from the scan raw data in a pixel-by-pixel manner as

(8) $$\begin{array}{c} \text{ADC}=\frac{\ln S_{1}-\ln S_{0}}{b_{1}-b_{0}}, \end{array}$$

where *b* characterizes the diffusion-sensitizing gradient pulses, with *b*_1_ = 1000 s/mm^2^ and *b*_0_ = 0 s/mm^2^ in our data. *S*_1_ is the diffusion-weighted signal intensity with *b*_1_ = 1000 s/mm^2^. *S*_0_ is the signal with no diffusion gradient applied, i.e., with *b*_0_ = 0 s/mm^2^.

The AIS lesions were manually annotated by two experienced experts (Dr. Song Jin and Dr. Chen Cao) from Tianjin Huanhu Hospital. The entire dataset includes 398 weakly labeled subjects and 184 fully labeled subjects, and they are divided into the training set and test set. The training set includes 398 weakly labeled subjects and 5 fully labeled subjects, which are used to train the network parameters. The test set includes the remaining 179 fully labeled subjects to evaluate the generalization capacities on unknown samples. For the sake of simplicity, we name the weakly labeled and fully labeled subjects in the training set as cla-data and seg-data, respectively.

As the MR images were acquired on the three different MR scanners, their matrix sizes are different, as shown in Table 1. Therefore, we resample all the MR images to the same size of 192 × 192 using linear interpolation. The pixel intensity of each MR image is normalized into that of zero mean and unit variance, and the DWI and ADC slices are channel-wise concatenated as dual-channel images and fed into the MFMF-Network. Data augmentation technique is adopted in both the classification process and the segmentation process. In particular, each input image is randomly rotated by a degree ranging from 1 to 360 degrees, flipped vertically and horizontally on the fly, so as to augment the dataset and reduce memory footprint.

### 3.2. Implementation Details

The parameters of the proposed MFMF-Network are shown in Figure 3. In the classification process, we initialize the main-pathway CNN using the pretrained parameters of VGG16 on ImageNet [16]. The FC layer parameters are initialized from zero-mean Gaussian distributions with a standard deviation of 0.1. After training the classification branch, we freeze the main-pathway CNN and initialize the other parameters in the segmentation branch, as suggested in [25]. In both the classification and segmentation processes, the RAdam method [26] with *β*_1_ = 0.9 and *β*_2_ = 0.999 is used as the optimizer and the initial learning rate is set as 10^−3^. The loss function used in this paper is binary cross-entropy (BCELoss).

We randomly select 0.1 of the cla-data as the validation set, which is used to fine-tune the hyperparameters in the classification process. During training, the learning rate is scaled down by a factor of 0.1 if no progress is made for 15 epochs on validation loss, and the training stops after 30 epochs with no progress on the validation loss. For the segmentation process, we pick all slices with lesions from the seg-data to train the segmentation branch. Dynamic learning rate scheduling is also adopted, where the learning rate is scaled down by a factor of 0.1 if no progress is made for 15 epochs on training loss. We stop the training of the segmentation process if the learning rate is 10^−9^ or no progress after 30 epochs on the training loss.

The experiments are performed on a computer with an Intel Core i7-6800K CPU, 64 GB RAM, and Nvidia GeForce 1080Ti GPU with 11 GB memory. The network is implemented in PyTorch. The MR image files are stored as Neuroimaging Informatics Technology Initiative (NIfTI) format and processed using Simple Insight ToolKit (SimpleITK) [27]. We use ITK-SNAP [28] for visualization.

### 3.3. Results

The proposed method is evaluated on the test set with 179 fully labeled subjects. For the sake of comparison, we also train and evaluate U-Net [29], FCN-8s [30], Res-UNet [21], and the method proposed in [31] on our dataset. For fairness consideration, the encoder parts of these methods are also pretrained as a classifier on our weakly labeled data. In particular, for the few-shot segmentation method proposed in [31], we split the slices of the seg-data with AIS lesions into the support set and query set. Other experimental details are the same as our proposed method except for freezing the pretrained parameters.

**Figure 4:**
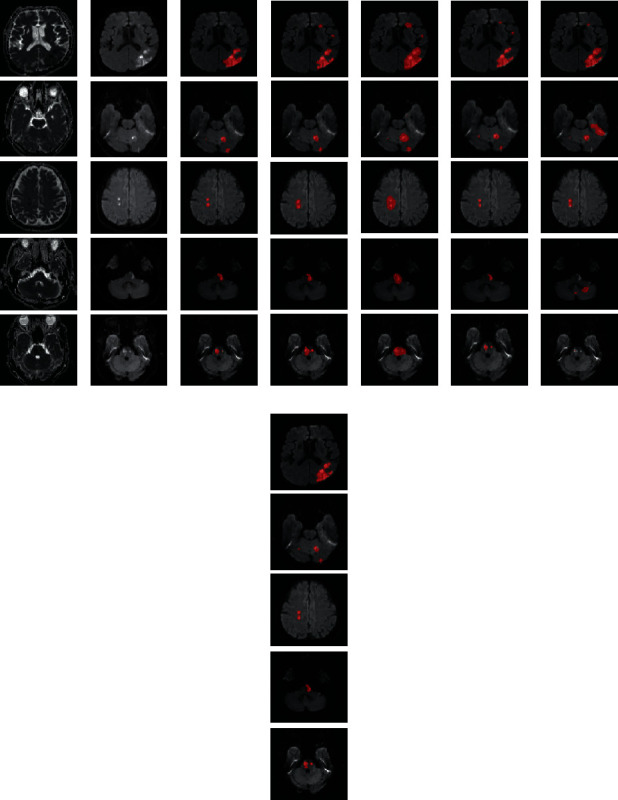
Visualization examples of the MRI slices and lesion segmentation results. (a–c) The original ADC map, DWI, and ground truth, respectively. (d–h) The segmentation results of U-Net, FCN-8s, Res-UNet, the method in [31], and the proposed method, respectively. The segmentation results are overlaid on the DWIs and highlighted in red.

Figure 4 visualizes some examples of AIS segmentation. As Figure 4 shows, our proposed method, i.e., column (h), is accurate on both the large and small AIS lesions. Even though U-Net and Res-UNet have more multifeature fusion, they overestimate the lesion but ignore the details of adjacent lesions. On the other hand, FCN-8s uses three-scale feature fusion, which is the same as our method, but the outputs of its last convolutional layer resampled to the size of input images require interpolation of 32 times, which inevitably leads to an overestimated lesion region. For the few-shot segmentation method proposed in [31], the multifeature fusion combines the support set with the query set to train the parameters. Nevertheless, the proportion of positive pixels in the medical slice is typically smaller than that of the natural image, making the few-shot segmentation method in [31] tend to ignore small lesions or misclassify the artifact regions as lesions, as shown in Figure 4.

The quantitative evaluation results are summarized in Table 2. As Table 2 shows, our proposed method achieves the best results on all of the metrics except for the recall rate. Specifically, our proposed method achieves a mean dice coefficient of 0.699 ± 0.128 from the aspect of the pixel-level metric, which is much higher than the results obtained by FCN-8s [30] and the few-shot segmentation method [31] and is also higher than that of U-Net [29] and Res-UNet [21]. For the lesion-wise metrics, our proposed method achieves the highest precision rate of 0.852 and the highest F1 score of 0.886 over the competitors. The recall rate of 0.923, however, is slightly worse than U-Net and FCN-8s due to the fact that they tend to cover a larger area than the real lesion size, which reduces the number of FNs when many small lesions gathered together. Furthermore, for the subject-wise metric, all of the methods achieve a detection rate of 1 except for the few-shot segmentation method in [31] and Res-UNet.

**Figure 5:**
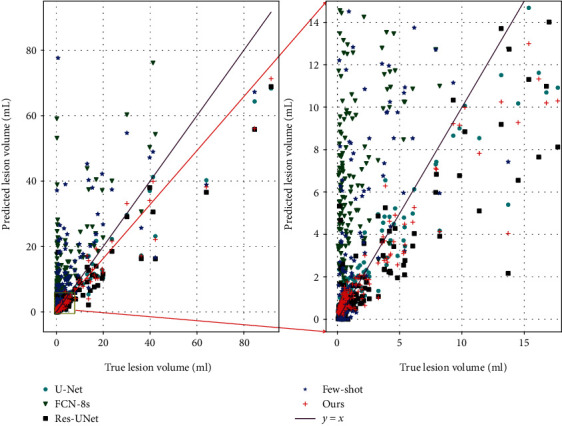
Predicted lesion volume versus ground truth volume.

Figure 5 further plots the scatter map between the volumes of the manual annotation and the predicted segmentation, where the purple line indicates a perfect match between the predicted volumes and the ground truth volumes. As Figure 5 shows, the predicted volumes of our proposed method are closer to the true volumes than the competitors.

## 4. Discussions

### 4.1. How Many Weakly Labeled Subjects Do We Need?

So far, we have shown that our proposed method can achieve high segmentation accuracy by using 398 weakly labeled and 5 fully labeled subjects. It is worth investigating whether we can further reduce the number of weakly labeled subjects. In particular, we randomly select proportions of 0.8, 0.6, 0.4, and 0.2 from the 398 subjects to train the classification branch.

Table 3 summarizes the evaluation results with different numbers of weakly labeled subjects. As we can see from Table 3, we can achieve a DR of 1 when more than 238 subjects are used to train the classification branch; besides, we can also achieve a higher mean dice coefficient and recall rate as the number of weakly labeled subjects increased. The other metrics, including the precision rate and F1 score, generally rise accompanied by small fluctuations.

### 4.2. Effect of Postprocessing

**Figure 6:**
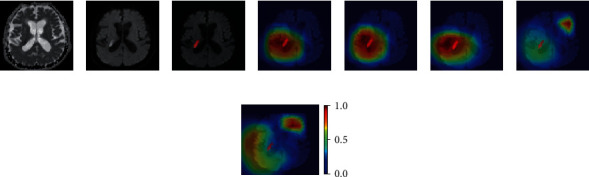
Examples of CAM. (a) ADC slice. (b) DWI slice. (c) Ground truth. (d) 398 subjects. (e) 318 subjects. (f) 238 subjects. (g) 159 subjects. (h) 79 subjects. The CAM and ground truth are depicted on the DWI. Best viewed in color.

From Table 3, we can also see that our proposed method uses 159 subjects to obtain the pretrained parameters achieving a detection rate of 0.966, which means that it fails to detect 6 subjects in the test set. In fact, the detection rate is 1 when the segmentation branch directly predicts the segmentation results without using postprocessing. However, the precision rate and the F1 score are much lower than those using postprocessing. To investigate the importance of postprocessing, we summarize the comparison results with different numbers of weakly labeled subjects, as shown in Table 4. As Table 4 shows, postprocessing greatly improves the dice coefficient, precision rate, and F1 score but reduces the detection rate, which is because of the CAM generated by the classification branch.  Figure 6 presents some samples of CAM. As Figure 6 shows, the CAM shows a higher probability in the suspected lesion region with the increasing number of weakly labeled subjects used in the classification branch. In particular, the CAM shows a probability of 0 or a probability below the threshold of *δ* = 0.5 in some subjects when less than 159 weakly labeled subjects are used to train the classification branch, which leads to missed diagnosis when postprocessing is used in the inference process. In a word, our postprocessing is critical for AIS lesion segmentation in this research.

### 4.3. Single Modal vs. Multimodal

In this subsection, we explore the effect of different modalities of MR images on our results. We use single-modal and multimodal subjects to train and test our proposed method. The dataset for training the classification branch includes all the 398 subjects regardless of the modal combination. As Table 5 shows, the multimodal subjects achieve the best results. The DWI also achieves competitive results compared with the multimodal. The DWI achieves competitive results due to the fact that the AIS lesions appear as hyperintense on the DWIs, which is more prominent to be recognized than that on the ADC maps. The combinational use of the DWI and ADC map, on the other hand, helps in reducing the FPs and FNs, which largely improves the segmentation results.

### 4.4. Impact of Using Lesion Slices Only

Note that we only extract slices with AIS lesions from the 5 fully labeled subjects in the seg-data to train the segmentation branch. In this subsection, we would like to further discuss whether the slices without any lesion should be included.  Table 6 summarizes the evaluation results after training on all subjects and only lesion slices. As Table 6 shows, the network trained on lesion slices shows superior performance over that trained on all slices on all metrics except the recall rate, which means that training on both the normal and lesion slices will reduce the number of FNs but increase the number of FPs. Intuitively, including the normal slices will make the class imbalance problem more severe, leading to inadequate learning on the lesion features. In fact, as the AIS lesion volume is much smaller than the normal tissues in most cases, the lesion slices have included much information about the normal tissue appearance. We can then conclude that to improve the segmentation accuracy, it is necessary to only include the lesion slices when training the segmentation branch.

### 4.5. Performance on Large and Small Lesions

Clinically, an AIS lesion is classified as a lacunar infarction (LI) lesion if its diameter is smaller than 1.5 cm [32]. LI is much difficult to be diagnosed in clinical practice, especially when it is too small to be noticed. Therefore, it is very necessary to evaluate the performance on LI.

In this subsection, we divide the test set into the small lesion set and large lesion set. A subject is categorized into a small lesion subject only if all of the lesions are LI lesions. Otherwise, it will be included in the large lesion set. In the test set, there are 118 subjects and 61 subjects included in the small lesion set and the large lesion set, respectively. As Table 7 shows, we achieve a mean dice coefficient of 0.718 ± 0.120 on the large lesion set, while a mean dice coefficient of 0.689 ± 0.222 on the small lesion set. On other metrics, our proposed method achieves higher performance on the small lesion set.

In clinical diagnosis, large lesions are more easily diagnosed, while small lesions are not. Our proposed method achieves high performance not only on large lesions but also on small lesions.

### 4.6. Performance on the Public Dataset

To demonstrate the effectiveness of the proposed method, the performance on an external public dataset is further evaluated. In particular, we choose to use the training set of SPES in the ISLES2015 challenge [33]. Even though the SPES task is originally designed for ischemic stroke outcome prediction, the training set includes the ADC maps (known as DWI in SPES) and the corresponding AIS lesion annotations. We randomly split the subjects in the SPES training set into three sets, i.e., training set, validation set, and test set, with 5, 5, and 20 subjects, respectively.

The classification branch is trained on our institutional weakly labeled images with 398 weakly labeled ADC subjects, and the segmentation branch is trained on the new training set and the validation set. By noting that the public dataset and our institutional dataset were acquired from various MRI scanners with different parameters, the statistical property varies, which is known as domain adaption. As the classification branch is trained on our institutional data, the threshold of CAM has to be further tuned by using the validation set to adapt the SPES data.

For the sake of comparison, we also train and evaluate the methods used in Section 3.3. For fairness consideration, the encoder parts of these methods are also pretrained as a classifier on our 398 weakly labeled ADC subjects. In particular, for the few-shot segmentation method proposed in [31], we split the slices of the new training set with AIS lesions into the support set and query set. Other experimental details are the same as used in Section 3.3 except that the validation loss determines when to stop the training.

**Figure 7:**
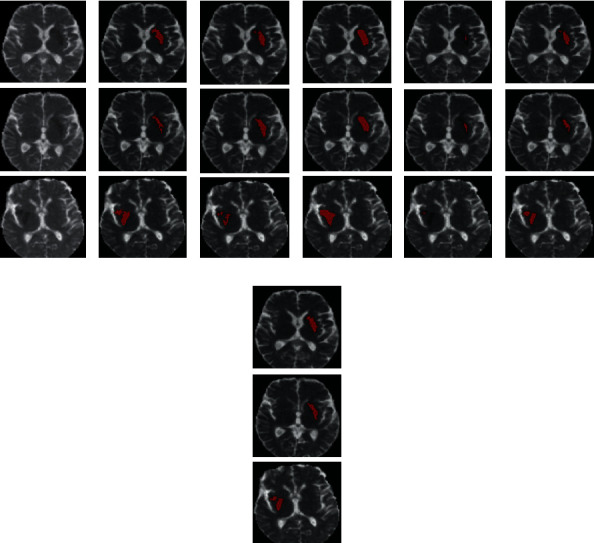
Visualization examples of the MRI slices and lesion segmentation results. (a, b) The original ADC map and ground truth, respectively. (c–g) The segmentation results of U-Net, FCN-8s, Res-UNet, the method in [31], and the proposed method, respectively. The segmentation results are overlaid on the ADCs and highlighted in red.

**Figure 8:**
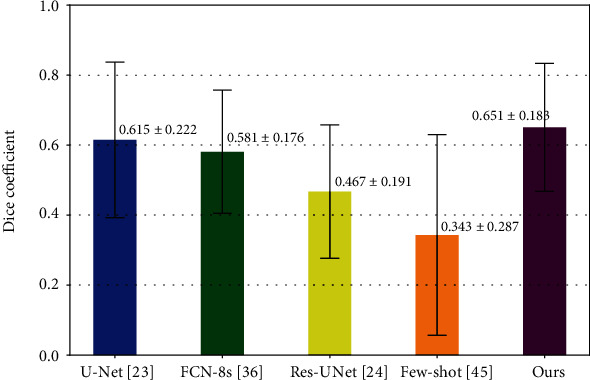
Bar plots of the dice coefficient for different methods.

Figure 7 plots some visualized examples on the test set. Similar to the results obtained on our institutional data, the proposed method achieves the best segmentation accuracy. As Figure 8 shows, the proposed method is able to achieve a mean dice coefficient of 0.651 ± 0.183, which highlights the better capacity of our proposed method even in the cross-domain case.

## 5. Conclusion

In this paper, we proposed a deep learning-based method using a few fully labeled subjects for AIS lesion segmentation. Our proposed method consists of three processes: classification, segmentation, and inference. Since there are no pretrained parameters available for processing medical images using CNN, some weakly labeled subjects are used to train the MFMF-Network to obtain a set of pretrained parameters in the classification process. Then, only 5 fully labeled subjects are used to train the segmentation branch.

The proposed method presents high performance on the clinical MR images with a mean dice coefficient of 0.699 ± 0.128 from the aspect of the pixel-level metric. More importantly, it presents a very high precision rate of 0.852 and recall rate of 0.923 from the lesion-wise metrics. Therefore, the proposed method can greatly reduce the expense of obtaining a large number of fully labeled subjects in a supervised setting, which is more meaningful in terms of engineering maneuverability.

## Figures and Tables

**Table 1:** Parameters used in DWI acquisition.

MR scanners	Skyra	Trio	Avanto
Repetition time (ms)	5200	3100	3800
Echo time (ms)	80	99	102
Flip angle (°)	150	120	150
Number of excitations	1	1	3
Field of view (mm^2^)	240 × 240	200 × 200	240 × 240
Matrix size	130 × 130	132 × 132	192 × 192
Slice thickness (mm)	5	6	5
Slice spacing (mm)	1.5	1.8	1.5
Number of slices	21	17	21

**Table 2:** Evaluation results on the test set. In particular, the mean DC is presented in the way of mean ± standard deviation. The best result has been highlighted in italic.

Method	DC	*P*_L_	*R*_L_	F1	DR
U-Net [29]	0.629 ± 0.152	0.285	*0*.*942*	0.437	1.000
FCN-8s [30]	0.289 ± 0.222	0.234	0.938	0.374	1.000
Res-UNet [21]	0.557 ± 0.227	0.494	0.901	0.638	0.972
Few-shot [31]	0.239 ± 0.253	0.191	0.591	0.288	0.642
Ours	*0.699* ± *0.128*	*0*.*852*	0.923	*0*.*886*	1.000

**Table 3:** Evaluation results obtained by using different numbers of weakly labeled subjects on the training set. The mean DC is presented in the way of mean ± standard deviation. The best result has been highlighted in italic.

Scale of the dataset	DC	*P*_L_	*R*_L_	F1	DR
79 subjects	0.557 ± 0.250	0.793	0.741	0.766	0.922
159 subjects	0.665 ± 0.181	*0*.*854*	0.872	0.863	0.966
238 subjects	0.675 ± 0.138	0.843	0.901	0.871	1.000
318 subjects	*0.700* ± *0.134*	0.821	0.920	0.867	1.000
398 subjects	0.699 ± 0.128	0.852	*0*.*923*	*0*.*886*	1.000

**Table 4:** Evaluation results by using different numbers of weakly labeled subjects with and without postprocessing. In particular, the mean dice coefficient is presented in the way of mean ± standard deviation.

Scale of the dataset	Postprocessing	DC	*P*_L_	*R*_L_	F1	DR
398 subjects	No	0.651 ± 0.158	0.403	0.956	0.567	1.000
318 subjects		0.649 ± 0.157	0.391	0.949	0.554	1.000
238 subjects		0.630 ± 0.165	0.383	0.949	0.546	1.000
159 subjects		0.593 ± 0.184	0.297	0.949	0.452	1.000
79 subjects		0.620 ± 0.209	0.487	0.898	0.632	0.979
398 subjects	Yes	0.699 ± 0.128	0.852	0.923	0.886	1.000
318 subjects		0.700 ± 0.134	0.821	0.920	0.867	1.000
238 subjects		0.675 ± 0.138	0.843	0.901	0.871	1.000
159 subjects		0.665 ± 0.181	0.854	0.872	0.863	0.966
79 subjects		0.557 ± 0.250	0.793	0.741	0.766	0.922

**Table 5:** Evaluation results of single-modal and multimodal MR images. The mean DC is presented in the way of mean ± standard deviation.

Modality	DC	*P*_L_	*R*_L_	F1	DR
ADC+DWI	0.699 ± 0.128	0.852	0.923	0.886	1.000
DWI	0.665 ± 0.166	0.743	0.876	0.804	0.989
ADC	0.451 ± 0.278	0.599	0.600	0.570	0.804

**Table 6:** Evaluation results of the MFMF-Network whose segmentation branch is trained on different data, where “all slices” means both the normal and lesion slices are used, and “lesion slices” means that only lesion slices are used. The best result has been highlighted in italic.

	DC	*P*_L_	*R*_L_	F1	DR
All slices	0.659 ± 0.124	0.702	*0*.*931*	0.801	1.000
Lesion slices	*0.699* ± *0.128*	*0*.*852*	0.923	*0*.*886*	1.000

**Table 7:** Evaluation results on large and small lesions. The best result has been highlighted in italic.

	DC	*P*_L_	*R*_L_	F1	DR
Large lesion set	*0.718* ± *0.120*	0.846	0.887	0.866	1.000
Small lesion set	0.689 ± 0.222	*0*.*858*	*0*.*962*	*0*.*907*	1.000

## Data Availability

The patient data used to support the findings of this study were supplied by Tianjin Huanhu Hospital, so they cannot be made freely available. The public dataset used in this paper is available at http://www.isles-challenge.org/ISLES2015/.
